# Urinary Insulin-Like Growth Factor-Binding Protein 7 (IGFBp7), Urinary Tissue Inhibitor of Matrix Metalloproteinase 2 (TIMP2), and Serum Transgelin as Novel Biomarkers of Kidney Injury in Multiple Myeloma

**DOI:** 10.1007/s12288-023-01701-x

**Published:** 2023-10-25

**Authors:** Sarah M. Shoeib, Asmaa Hassan, Eman Habeeb, Rasha Abdallah Ragab, Sara Elakshar, Dalia Sherief

**Affiliations:** 1 Department of Clinical Pathology, Faculty of Medicine, Tanta University, Tanta, Egypt; 2 Department of Clinical Pathology, Faculty of Medicine, Kafr El-Sheikh University, Kafr El-Sheikh, Egypt; 3 Department of Internal Medicine, Faculty of Medicine, Kafr El-Sheikh University, Kafr El-Sheikh, Egypt; 4 Department of Clinical Oncology and Nuclear Medicine, Faculty of Medicine, Tanta University, Tanta, Egypt

**Keywords:** Multiple myeloma, Renal impairment, IGFBP-7, TIMP-2, Transgelin

## Abstract

Renal dysfunction is a common complication of MM and is associated with poor prognosis, particularly when progressive. Early identification of renal dysfunction is essential for prompt treatment for disease control and restoration of renal function. Urinary insulin-like growth factor-binding protein 7 (IGFBP-7), urinary tissue inhibitor of matrix metalloproteinase 2 (TIMP-2), and serum transgelin levels were measured using enzyme-linked immunosorbent assays and evaluated as biomarkers for the prediction of renal impairment in patients with multiple myeloma. U _IGFBP-7/creatinine_ ratio, U _TIMP2/creatinine_ ratio, and serum transgelin levels were higher in patients with MM than healthy controls, and predicted renal insufficiency in MM. Serum transgelin, urinary IGFBp7, and TIMP2 levels may have utility as biomarkers of renal tubular injury and predict future renal impairment in patients with MM.

## Introduction

Multiple myeloma is a neoplastic disease of plasma cells that is prevalent in older individuals. Bone pain and pathological fractures due to osteolytic bone lesions, hypercalcemia, renal insufficiency, and bone marrow failure are the primary clinical features of MM [1].

The three new criteria recently been added to the diagnostic requirements of multiple myeloma by the International Myeloma Working Group (IMWG) are: 60% of marrow plasma cells are monoclonal; a free light chain (FLC) ratio of greater than 100 if the implicated serum FLC level is equal or greater than 100 mg/L,; and multiple localized lesions on magnetic resonance imaging (MRI) [2].

Approximately 25% of MM patients develop renal dysfunction which is associated with poor prognosis, particularly if renal dysfunction is persistent or progressive. However, the early and accurate identification of patients at risk of renal impairment is challenging using routine laboratory tests such as serum creatinine and estimated glomerular filtration rate [3].

As renal injury is a poor prognostic factor and increases the risk of future problems in patients with multiple myeloma, early identification is essential to allow prompt treatment for disease control and restoration of renal function [4]. Histological analysis of renal biopsy specimens remains the gold standard technique for the diagnosis of MM-related renal dysfunction; however, the significant morbidity and mortality associated with renal biopsy preclude its use as a screening tool. Accordingly, there is increasing interest in the development of non-invasive, accurate, and applicable diagnostic markers based on the pathogenesis of MM-related kidney disease [5].

The measurement of urinary levels of tissue inhibitor of matrix metalloproteinase 2 (TIMP-2) and insulin-like growth factor-binding protein 7 (IGFBP7), both function in promoting G1 cell cycle arrest, has demonstrated utility in predicting acute kidney injury [1, 6]. Additionally, TIMP-2 has utility as a marker of injury to distal renal tubular cells, while proximal cells have been shown to secrete IGFBP-7[1].

Transgelin-2 (SM22), a cytoskeletal actin-binding protein, is expressed by stem cells, fibroblasts, immune cells, and certain epithelial cells. Transgelin has also been shown to have utility as a marker of renal injury, glomerulosclerosis, and interstitial fibrosis [7]. Increased expression of transgelin has been observed in tubulointerstitial injury and glomerular injury, and is posited to be tissue-specific depending on the underlying disease etiology [1].

The study’s objective was to evaluate the utility of urinary IGFBP-7, urinary TIMP-2, and serum transgelin levels as biomarkers for the prediction of renal impairment in patents with multiple myeloma.

## Materials and Methods

### Study Design

This was a prospective study comprising a total of 90 patients attending the hematology and oncology units at Tanta and Kafr El Sheikh University Hospitals from July 2020 to January 2021. All patients had been diagnosed with multiple myeloma according to IMWG criteria. Exclusion criteria were recent infectious illness, hepatitis B virus infection, hepatitis C virus infection, AIDS, and neoplasms other than myeloma.

The control group comprised 30 healthy participants who were age- and sex-matched relatives of patients who were free from chronic or acute illness. Reference values used for non-routine laboratory tests in the present study were based on values from the control group.

### Data Collection

The following sociodemographic and clinical data were retrieved from medical records in all patients: age, sex, date of initial diagnosis, and treatment protocol. We also used laboratory reports to obtain urinary light chain concentrations and serum levels of creatinine, albumin, beta 2-microglobulin, monoclonal protein, and creatinine. The Chronic Kidney Disease Epidemiology Collaboration (CKD-EPI) 2009 formula was used to calculate estimated glomerular filtration rate based on serum creatinine [8].

### Measurement of Serum Transgelin, and Urinary IGFBP-7, and TIMP-2 Levels

Serum transgelin and urinary IGFBP-7 and TIMP-2 levels were measured in urine and serum samples taken from patients and control groups under strict aseptic conditions. All samples were processed and then stored at a temperature below 20° C. Serum transgelin levels were quantified using ELISA kits for transgelin from Lifespan BioSciences, Inc (catalog no: LS-F12693). Human IGFBP7 ELISA kits from Abcam was used to measure urinary IGFBP-7 levels (catalog no: ab229894). Quantikine ELSA Human TIMP-2 Immunoassays were used to measure urinary TIMP-2 levels (R &D systems, Catalog Number DTM200). All urinary biomarker levels were normalized by dividing by urine creatinine.

Transgelin, IGFBP-7, and TIMP-2 were chosen due to the availability, reasonable price, and simple methodology of respective ELISA techniques.

### Patient Follow-up

Follow up information was gathered 24 months later at the final follow-up visit from medical records including treatment response, date and reason of death, and laboratory tests results including serum creatinine and eGFR. Patients were divided into four categories according to response to treatment as follows: complete response, partial response, steady disease (SD), or progressing disease (PD).

### Data Processing

All data were evaluated using IBM SPSS software package version 20.0.

## Results

*Patient characteristics* The present study comprised 90 patients with multiple myeloma and 30 healthy individuals matched in terms of age and gender as a control group. No statistically significant difference in age or gender were observed between groups (Table 1).

**Table 1:** Comparison between the two groups according to different parameters

	Patients (n = 90)	Control (n = 30)	Test of sig	*P*
*Sex*				
Male	40(44.4%)	16(53.3%)	χ^2^ = 0.714	0.398
Female	50(55.6%)	14(46.7%)		
*Age (years)*				
Mean ± SD	68 ± 5.1	66.4 ± 5.3	t = 1.535	0.127
Median (Min.–Max.)	68(60–79)	64(58–76)		
*U *_*IGFBp7/Creatinine*_				
Mean ± SD	0.71 ± 0.49	0.06 ± 0.02	U = 82.0*	< 0.001*
Median (Min.–Max.)	0.68 (0.04–2.07)	0.06 (0.03–0.10)		
*U *_*TIMP2/Creatinine*_				
Mean ± SD	0.17 ± 0.12	0.02 ± 0.01	U = 137.0*	< 0.001*
Median (Min.–Max.)	0.17 (0.01–0.44)	0.01 (0.01–0.03)		
*Serum transgelin*				
Mean ± SD	79.54 ± 11.05	63.28 ± 7.65	t = 8.937*	< 0.001*
Median (Min.–Max.)	83 (60–99)	64.10 (50.2–80)		

*t* Student t-test; *U* Mann Whitney test; *χ*^*2*^ Chi square test

P value <0.05 are considered significant

*Results of the novel markers of kidney injury* The novel biomarkers of tubular injury were assessed in the two groups and their concentrations were considerably higher in the MM patients investigated than in the healthy controls (*P* < 0.001*) as shown in (Table 1).

*Comparison of kidney injury markers in patients with different stages of myeloma* Patients were subdivided into three groups according to the International Staging System (ISS) for multiple myeloma: stage I (12 patients), stage II (48 patients) and stage III (30 patients). Statistically significant differences in urinary IGFBp7/ urinary creatinine (U _IGFBP-7/creatinine_) and urinary TIMP2/ urinary creatinine (U _TIMP2/creatinine_) ratios were observed between groups (*P* = 0.015 and *P* = 0.015 respectively). On the other hand, no statistically significant differences in serum transgelin levels were observed between groups (*P* = 0.058; Table 2).

**Table 2:** Comparison of the studied biomarkers in patients with different stages of myeloma (n = 90)

	Stage			Test of sig	*p*
	I (n = 12)	II (n = 48)	III (n = 30)		
*U *_*IGFBp7/ Creatinine*_					
Mean ± SD	0.37 ± 0.39	0.66 ± 0.40	0.93 ± 0.57	H = 9.305*	0.010*
Median (Min.–Max.)	0.17 (0.05–1.06)	0.71 (0.05–1.67)	0.73 (0.04–2.07)		
*U *_*TIMP2/Creatinine*_					
Mean ± SD	0.08 ± 0.07	0.18 ± 0.11	0.19 ± 0.13	H = 8.369*	0.015*
Median (Min.–Max.)	0.05 (0.02–0.22)	0.20 (0.01–0.40)	0.15 (0.01–0.44)		
*Serum transgelin*					
Mean ± SD	72.60 ± 10.89	80.17 ± 12.10	81.3 ± 8.3	F = 2.946	0.058
Median (Min.–Max.)	68.45 (60–88.20)	85.35 (60–99)	83.0 (60.0–92.0)		

*F* F for One way ANOVA test; *H* H for Kruskal Wallis test

P value <0.05 are considered significant

*Comparison of kidney injury markers in patients with different eGFR* Among all patients, 53 patients had an eGFR of <60 mL/min/1.73 m^2^ and 37 patients had an eGFR of ≥ 60 mL/min/1.73 m^2^. Statistically significant differences in U _IGFBP-7/creatinine_ and U _TIMP2/creatinine_ ratios were observed between groups (*P* =  < 0.001 and *P* = 0.006 respectively). On the other hand, no statistically significant differences in serum transgelin levels were observed between groups (*P* = 0.959; Table 3).

**Table 3:** Comparison of kidney injury markers in patients with different eGFR: (n = 90)

	eGFR (CKD-EPICr)		Test of sig	*p*
	<60 mL/min/1.73 m^2^. (n = 53)	≥ 60 mL/min/1.73 m^2^. (n = 37)		
*U *_*IGFBp7/Creatinine*_				
Mean ± SD	0.87 ± 0.54	0.48 ± 0.30	U = 558.50*	< 0.001*
Median (Min.–Max.)	0.78 (0.05–2.07)	0.50 (0.04–0.89)		
*U *_*TIMP2/Creatinine*_				
Mean ± SD	0.20 ± 0.13	0.12 ± 0.08	U = 640.50*	0.006*
Median (Min.–Max.)	0.20 (0.02–0.44)	0.14 (0.01–0.24)		
*Serum transgelin*				
Mean ± SD	79.49 ± 10.16	79.61 ± 12.43	t = 0.052	0.959
Median (Min.–Max.)	81.50 (60–94.30)	84.50 (60–99)		

*t* Student t-test; *U* Mann Whitney test

P value <0.05 are considered significant

*Correlation of the studied biomarkers with established markers of renal injury* U _IGFBP-7/creatinine_ and U _TIMP2/creatinine_ ratios were significantly inversely correlated with serum albumin and eGFR while serum transgelin was significantly inversely correlated with serum albumin only. Also, there were significant positive correlation between U _IGFBP-7/creatinine_ and U _TIMP2/creatinine_ ratios and B2 microglobulin and serum creatinine. To the contrary, serum transgelin, did not correlate significantly with B2 microglobulin nor serum creatinine (Table 4).

**Table 4:** Correlation of the studied biomarkers with other established markers of renal injury

	N	U _IGFBp7/creatinine_		U _TIMP2/creatinine_		Serum transgelin	
		r_s_	*P*	r_s_	*p*	R	*P*
Albumin	90	*− 0.449*	< 0.001^*^	*− 0.320*	0.002^*^	*− 0.217*	0.040^*^
B2 microglobulin	90	*0.314*	0.003^*^	*0.197*	*0.063*	0.205	0.053
eGFR	90	*− 0.591*	< 0.001^*^	*− 0.512*	< 0.001^*^	− 0.169	0.111
Serum creatinine	90	*0.544*	< 0.001^*^	*0.462*	< 0.001^*^	0.143	0.180

P value <0.05 are considered significant

**Fig. 1:**
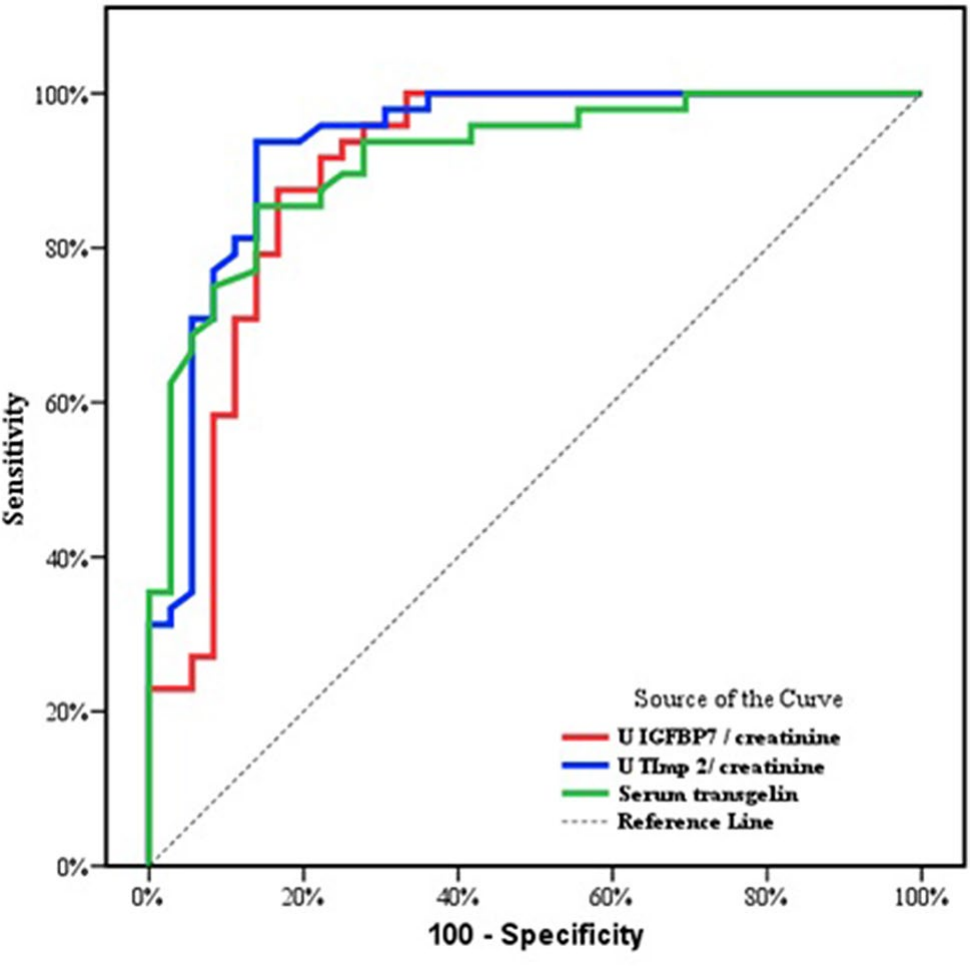
ROC curve for various markers to predict renal insufficiency (eGFR < 60 mL/min/1.73 m^2^ after therapy)

*Evaluation of baseline urinary markers and baseline serum transgelin as a predictor of kidney function after a follow-up of 24 months from the start of the study* At the end of the follow-up period, it was found that patients with an eGFR < 60 mL/min/1.73 m^2^ had higher levels of the studied biomarkers (when measured at the beginning of the study) than patients with an eGFR of ≥ 60 mL/min/1.73 m^2^. ROC curve analysis demonstrated that U _IGFBP-7/creatinine_ ratio, U _TIMP2/creatinine_ ratio, and serum transgelin had predictive value for renal insufficiency (eGFR < 60mL/min/1.73 m^2^). The area under the curve (AUC) values were 0.898, 0.934, and 0.915, respectively, and sensitivities of 87.50%, 93.75%, and 85.42%, respectively (Fig. 1 and Table 5).

**Table 5:** Prognostic performance for different markers to predict renal impairment (eGFR < 60 mL/min/1.73 m^2^) after therapy

	AUC	P	95% C.I	Cut off	Sensitivity	Specificity	PPV	NPV
U _IGFBp7/Creatinine_	0.898	< 0.001*	0.823–0.973	> 0.582	87.50	83.33	87.5	83.3
U _TIMP2/Creatinine_	0.934	< 0.001*	0.877–0.991	> 0.127	93.75	86.11	90.0	91.2
Serum transgelin	0.915	< 0.001*	0.855–0.975	> 79.5	85.42	86.11	89.1	81.6

*AUC* Area under a curve, *p value* Probability value, *CI* Confidence intervals, *NPV* Negative predictive value, *PPV* Positive predictive value

P value <0.05 are considered significant

**Fig. 2:**
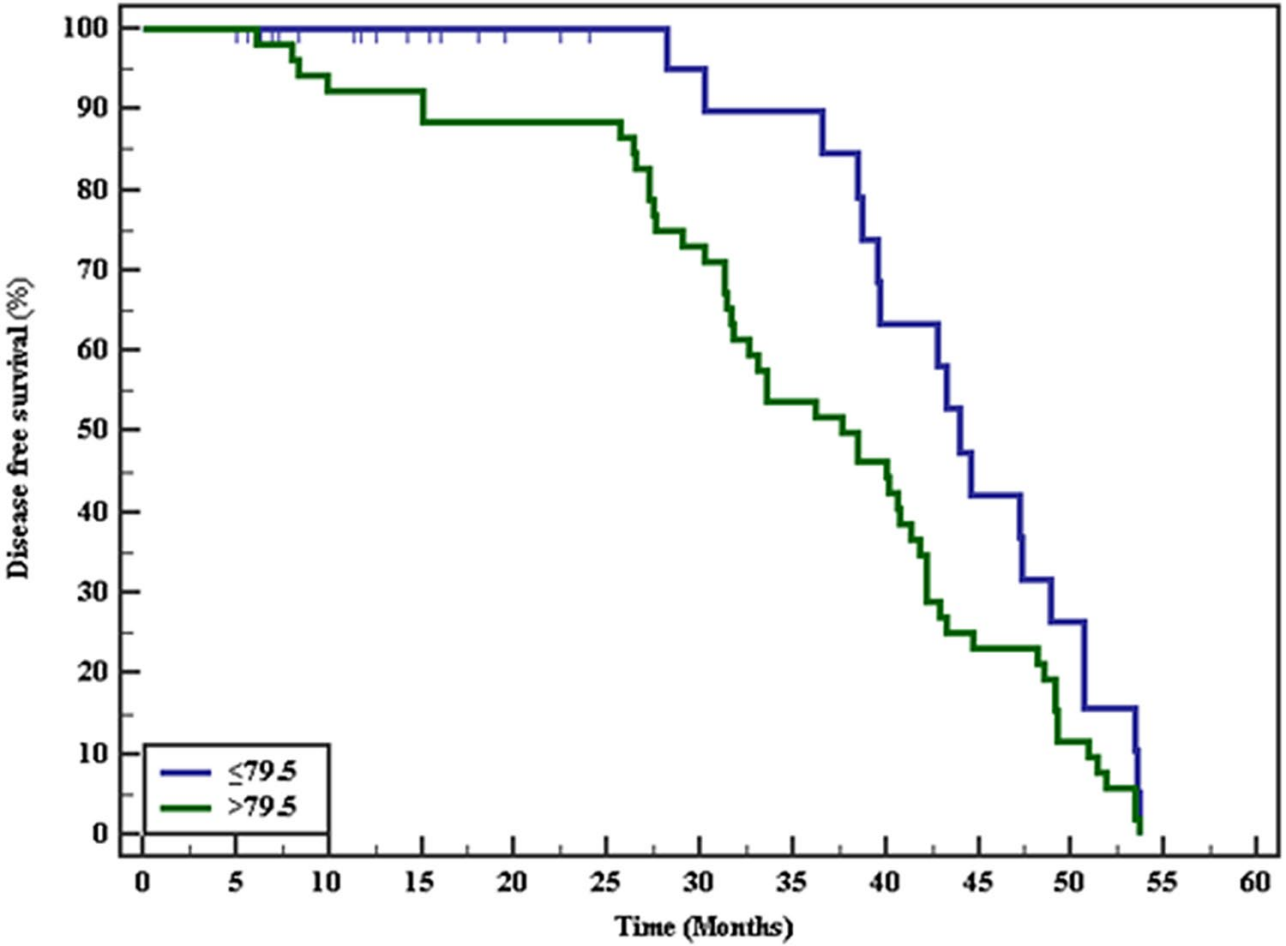
Kaplan–Meier survival curve for disease free survival with serum transgelin

**Fig. 3:**
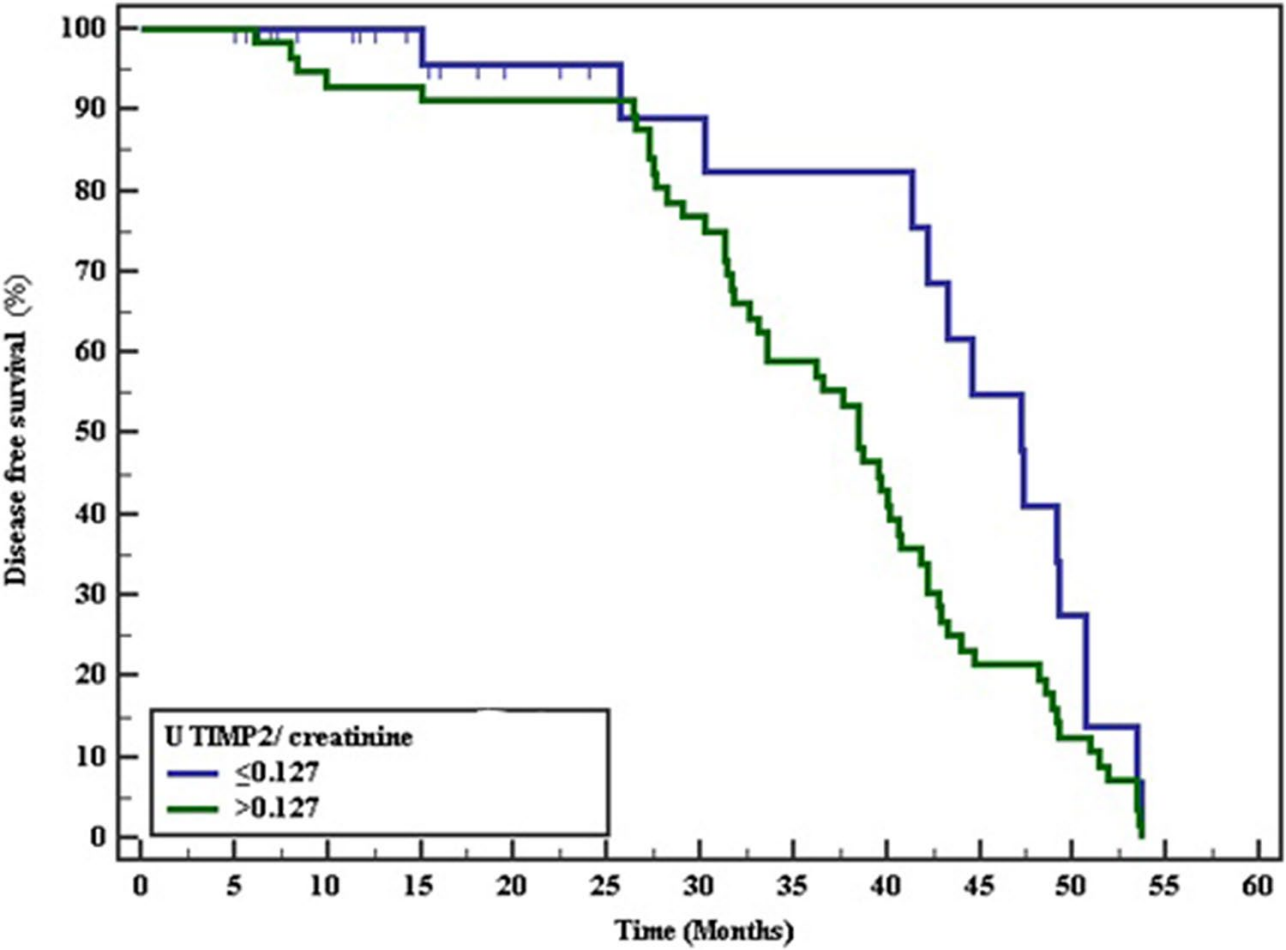
Kaplan–Meier survival curve for disease free survival with U _TIMP2/creatinine_

**Fig. 4:**
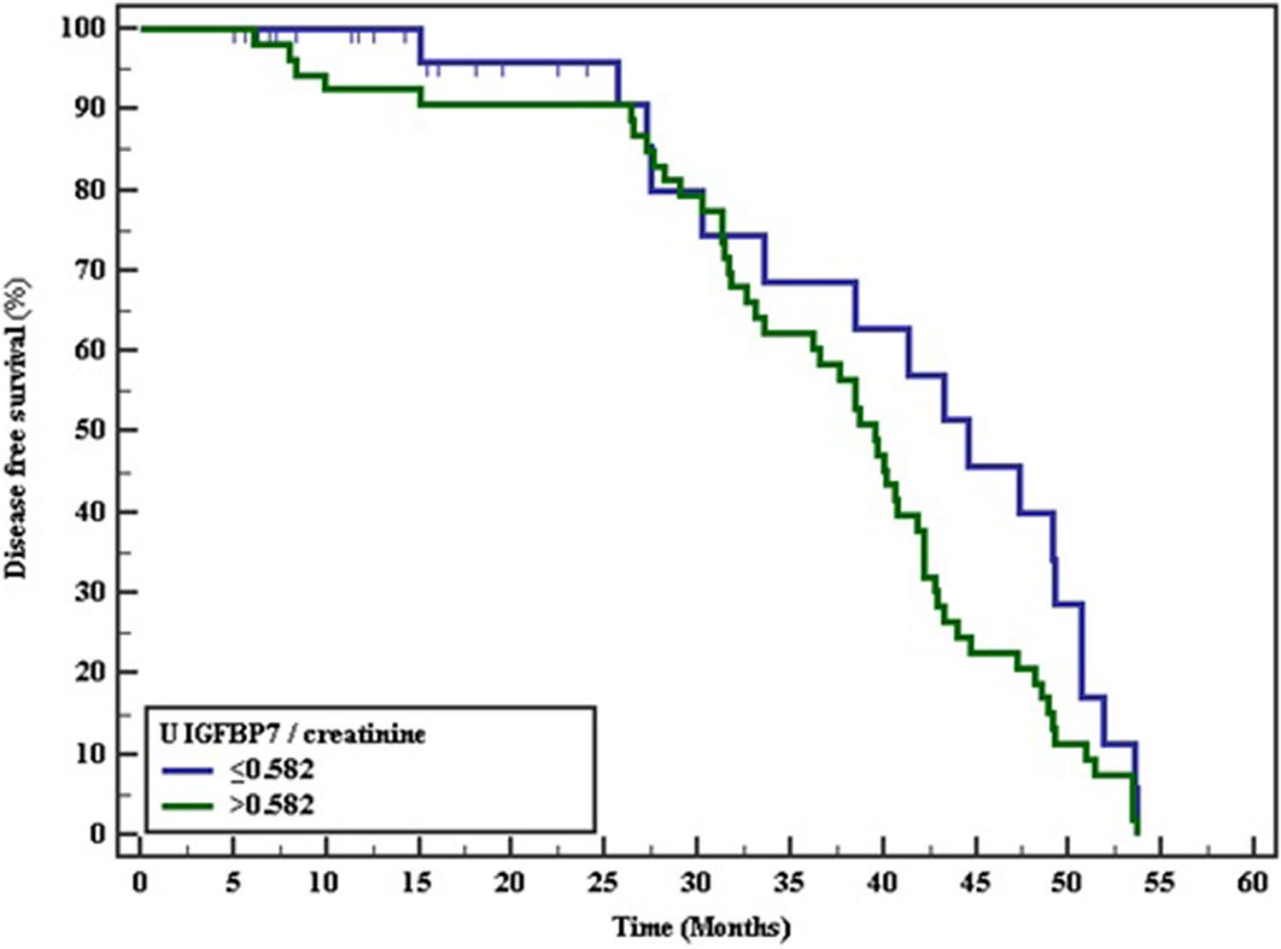
Kaplan–Meier survival curve for disease free survival with U _IGFBp7/ creatinine_

*Associations between kidney injury markers and survival* The associations between kidney injury markers and disease-free survival were assessed using Kaplan–Meier survival curves. Patients with serum transgelin levels of ≤ 79.5 and urinary U _TIMP2/creatinine_ ratio ≤ 0.127 had longer survival than other patients. (Figs. 2, 3 and Table 6); however, U _IGFBP-7/creatinine_ ratio was not associated with survival (Fig. 4, and Table 6).

**Table 6:** The Kaplan–Meier survival curve for Disease free survival

	Mean	% 12 Months	% 24 Months	% 36 Months	% 48 Months	% End of study	Log rank	
							χ^2^	*p*
*Serum transgelin*								
≤ 79.5	43.806	100.0	100.0	89.7	31.7	0.0	3.880*	0.049*
> 79.5	35.797	92.3	88.5	53.8	23.1	0.0		
*U *_*IGFBp7/creatinine*_								
≤ 0.582	40.487	100.0	96.0	68.6	40.0	0.0	3.180	0.075
> 0.582	36.933	92.0	90.6	62.3	20.8	0.0		
*U *_*TIMP2/creatinine*_								
≤ 0.127	43.639	100.0	95.5	95.5	41.1	0.0	4.503*	0.034*
> 0.127	36.593	92.9	91.1	91.1	21.4	0.0		

P value <0.05 are considered significant

## Discussion

The present study was to evaluate the association of the serum transgelin-2, urinary IGFBp7, and urinary TIMP2, with renal impairment over a 24-month monitoring period in individuals with MM. Higher initial serum transgelin levels, urinary IGFBp7 and urinary TIMP2 had predictive value in detecting renal impairment at the end of the study period regardless of initial eGFR.

In our study cohort, the concentrations of U _IGFBP-7/creatinine_ ratio, U _TIMP2/creatinine_ ratio and serum transgelin levels were considerably higher in patients with MM compared to healthy controls. These findings corroborate the results of Woziwodzka et al. [1, 9].

TIMP-2 contributes to carcinogenesis and the development of MM by promoting tumor cell proliferation and metastasis [10]. However, Zakiyanov et al. and Mora-Gutiérrez et al. reported increased TIMP-2 levels in a number of nephropathies, including diabetes, vasculitis, tubulointerstitial fibrosis, and glomerulosclerosis [10, 11]. TIMP-2 is predominantly synthesized by the distal tubule, however, extrarenal production of TIMP-2 has been linked to the development of myeloma [12].

Transgelin expression is up-regulated in several cancer types indicating possible role in oncogenesis and shown to promote the proliferation and differentiation of mesenchymal stem cells from bone marrow. Further, transgelin is reportedly up-regulated in leukaemia and lymphoma cells [13].

This finding supports the work of Woziwodzka et al. who posited serum transgelin levels are involved in carcinogenesis and the emergence of cancer and may differ depending on the disease stage and tumor size. TIMP-2 has been shown to regulate NF-kB signaling, with higher TIMP-2 levels observed in inflammation (i.e., SIRS), and function as a vascular inflammatory modulator [14].

We observed statistically significant differences in U _IGFBP-7/creatinine_ and U _TIMP2/creatinine_ ratios between groups categorized according to MM disease stage in the present study. However, no statistically significant differences in serum transgelin levels were observed between MM disease stages. This finding corroborates the results of Woziwodzka et al. who reported positive correlations for urinary levels of IGFBP-7 and TIMP2 of with ISS stage. They also observed that the majority of patients with ISS stages II and III had higher urinary levels of IGFBP-7 than healthy individuals [9].

In the present study, individuals with an eGFR of < 60 mL/min/1.73 m^2^ had comparable U _IGFBP-7/creatinine_, and U _TIMP2/creatinine_ ratios to patients with higher eGFR values but not with serum transgelin. Woziwodzka et al. reported no significant differences in urinary TIMP2 between patients with an eGFR of < 60 mL/min/1.73 m^2^ and patients with an eGFR of ≥ 60 mL/min/1.73 m^2^ [9]. Urbaniak-Kujda et al. posited TIMP-2 contributes to carcinogenesis, disease progress, and the development of osseous lesions in a study evaluated the utility of TIMP-2 as a a biomarker for kidney injury in myeloma [15]. Indeed, increased TIMP-2 production in stromal cells of the bone marrow may be essential for the development of osteolytic lesions in MM possible due to a weaker association betweenTIMP-2 levels and eGFR [16].

Woziwodzka et al. reported a significant increase in urinary IGFBP-7 levels in patients with an eGFR less than 60 mL/min/1.73 m^2^. Dittmann et al. also observed an association between serum levels of insulin-like growth factor-binding proteins and decreased eGFR [17].

The findings of the present study demonstrate that U _IGFBP-7/creatinine_ and U _TIMP2/creatinine_ ratios and serum transgelin levels have predictive value for renal insufficiency, a finding also reported by Woziwodzka et al. According to previous studies, the measurement of urinary NGAL levels may have utility as a more accurate predictor of RI in MM than serum levels as patients with renal involvement have higher urinary levels of NGAL and urinary FLC levels which are associated with eGFR [18].

In the present study, a significant association between serum transgelin levels and U _TIMP2/creatinine_ ratio and disease-free survival were observed, however, U _IGFBP-7/creatinine_ ratio was not associated with disease-free survival, these findings are inconsistent with the results of Bolomsky et al., who observed poor survival in patients with high urinary levels of IGFBp7 and TIMP2 and chromosomal abnormalities in MM cells with increased IGFBP-7 expression, an indicator of high-risk disease [19].

Our findings support a previous study by Yin et al. that posited transgelin-2 may represent as a therapeutic target given the specific expression of transgelin-2 by tumor cells. Transgelin-2 overexpression has also been shown to be associated with poor prognosis [20].

The combination of lenalidomide, dexamethasone, and bortezomib (a proteasome inhibitor) is the most common three-drug combination used to treat MM. Myeloma cells can be targeted by specific monoclonal antibodies (such as daratumumab, elotuzumab, isatuximab, and belantamab mafodotin), nuclear export inhibitors (such as selinexor), or histone deacetylase inhibitors (such as panobinostat) in cases of recurrent or treatment-resistant MM. Bortezomib is considered one of the most significant novel therapies for MM due to its numerous anti-myeloma effects including interruption of the cell cycle and activation of apoptosis, changes in the microenvironment of the bone marrow, and suppression of nuclear factor kappa B (NFB).

Patients with MM renal dysfunction may particularly benefit for treatment with bortezomib given its effect on improving renal function [21]. Bolomsky et al. also observed an association between treatment response to lenalidomide-dexamethasone and gene expression levels in bone marrow mononuclear cells [22].

The present study had several limitations. The present study comprised established cases of MM rather than patients with new diagnoses. Further, there were differences in sociodemographic and clinical characteristics between groups, patients were exposed to varying treatment plans, and a small sample size. Further studies using kidney biopsy samples may further elucidate the relationships of serum transgelin, urinary IGFBp7, and TIMP2 levels with renal dysfunction in MM. Additionally, studies comparing cases of MM renal involvement to a control group are required to validate the findings of the present study.

## Conclusions

In individuals with MM, serum transgelin, U _IGFBP-7/creatinine_ and U _TIMP2/creatinine_ ratios may have utility as biomarkers of predicting advanced chronic kidney disease stages.

Increased urinary IGFBp7 and TIMP2 levels in advanced stages of MM suggest that they may play a role in disease staging.

Early diagnosis using these biomarkers may lead to improve renal outcomes and increase life expectancy in MM.

## Data Availability

By contacting the respective author, data are available.
